# The Effect of Inflammatory Priming on the Therapeutic Potential of Mesenchymal Stromal Cells for Spinal Cord Repair

**DOI:** 10.3390/cells10061316

**Published:** 2021-05-25

**Authors:** Inés Maldonado-Lasunción, Agnes E. Haggerty, Akinori Okuda, Tokumitsu Mihara, Natalia de la Oliva, Joost Verhaagen, Martin Oudega

**Affiliations:** 1 The Miami Project to Cure Paralysis, University of Miami Miller School of Medicine, Miami, FL 33136, USA; ahaggerty@akoyabio.com (A.E.H.); okuda74@icloud.com (A.O.); mihara551231@tottori-u.ac.jp (T.M.); natalia.oliva.munoz@gmail.com (N.d.l.O.); 2 Department of Regeneration of Sensorimotor Systems, Netherlands Institute for Neuroscience, Institute of the Royal Netherlands Academy of Arts and Sciences, 1105 BA Amsterdam, The Netherlands; jverhaagen@nin.knaw.nl; 3 Biologics Lab, Shirley Ryan Ability Lab, Chicago, IL 60611, USA; 4 Department of Physical Therapy and Human Movement Sciences, Northwestern University, Chicago, IL 60611, USA; 5 Department of Orthopedic Surgery, Nara Medical University, Nara 634-8522, Japan; 6 Department of Orthopedic Surgery, Faculty of Medicine, Tottori University, Tottori 683-8504, Japan; 7 Department of Physiology, Northwestern University, Chicago, IL 60611, USA; 8 Edward Hines Jr. VA Hospital, Hines, IL 60141, USA

**Keywords:** macrophages, MSC, cell therapy, SCI, contusion, repair, neuroprotection, angiogenesis, immunomodulation

## Abstract

Mesenchymal stromal cells (MSC) are used for cell therapy for spinal cord injury (SCI) because of their ability to support tissue repair by paracrine signaling. Preclinical and clinical research testing MSC transplants for SCI have revealed limited success, which warrants the exploration of strategies to improve their therapeutic efficacy. MSC are sensitive to the microenvironment and their secretome can be altered in vitro by exposure to different culture media. Priming MSC with inflammatory stimuli increases the expression and secretion of reparative molecules. We studied the effect of macrophage-derived inflammation priming on MSC transplants and of primed MSC (pMSC) acute transplants (3 days) on spinal cord repair using an adult rat model of moderate–severe contusive SCI. We found a decrease in long-term survival of pMSC transplants compared with unprimed MSC transplants. With a pMSC transplant, we found significantly more anti-inflammatory macrophages in the contusion at 4 weeks post transplantation (wpt). Blood vessel presence and maturation in the contusion at 1 wpt was similar in rats that received pMSC or untreated MSC. Nervous tissue sparing and functional recovery were similar across groups. Our results indicate that macrophage-derived inflammation priming does not increase the overall therapeutic potential of an MSC transplant in the adult rat contused spinal cord.

## 1. Introduction

Mesenchymal stromal cells (MSC) respond to injury by secreting a combination of molecules that can support repair of different types of tissue, including central nervous tissue [1,2,3,4,5]. For this ability, transplantation of MSC has been widely applied as a strategy to stimulate tissue repair [6,7,8,9,10,11]. The injured spinal cord lacks the ability to spontaneously repair itself and is characterized by chronic unresolved inflammation [12,13,14] and secondary nervous tissue loss [15]. A transplant of unprimed, i.e., naïve, MSC supports immunomodulation [7] and nervous tissue sparing [6,16] in the adult contused rat spinal cord. The repair potential of MSC transplants is recognized, but their repair effects remain limited, which restricts clinical translation. Therefore, strategies to enhance the therapeutic efficacy of MSC transplants are pursued.

The composition and availability of the secretome of transplanted MSC determines their reparative effects and are governed by the local microenvironment [17,18,19]. One approach to modify MSC’s phenotype in order to acquire a more reparative secretome is priming, i.e., preconditioning, the cells prior to transplantation into injured tissue [20]. Priming approaches for MSC transplants have been tested for different applications, e.g., inflammatory diseases [21,22,23] and cardiomyopathies [24,25,26], resulting in therapeutic improvements. Recently, we investigated the potential of macrophage-derived inflammatory medium to prime MSC transplants [27]. We found that exposure of MSC to medium from pro-inflammatory macrophages causes an increase in expression and secretion of molecules known to support tissue repair [22,27,28], while also inducing the down regulation of genes associated with MSC survival [27].

In the present study, we explored the ability of inflammation primed MSC to support repair of the damaged spinal cord and the effect of inflammatory priming on transplant survival in vivo. For this, we used a clinically relevant rat model of spinal cord contusion. We investigated the effects of primed MSC transplants on nervous tissue sparing, immunomodulation, revascularization, and functional recovery of the contused spinal cord.

## 2. Materials and Methods

### 2.1. Animals 

Adult Sprague–Dawley female rats (*n* = 66, 225–250 g, Charles Rivers Laboratory, Wilmington, MA, USA) were used in this study. Additional rats of the same type (*n* = 8) were used to obtain bone-marrow-derived primary cell cultures of monocytes and MSC. All animal procedures were performed at the Miami Project to Cure Paralysis at University of Miami, Miami, FL, following the guidelines of the National Institutes of Health and the United States Department of Agriculture. The local Institutional Animal Care and Use Committee (IACUC) approved the procedures and the Assessment and Accreditation of Laboratory Animal Care accredited the animal facility where they took place. The animal study protocol used for these experiments was #17-087, approved by IACUC on May 4th, 2017. Rats were housed in pairs under a 12-hour light/dark cycle with ad libitum access to food and water.

### 2.2. Pre- and Post-Surgery Procedures 

Before surgical procedures, rats were anesthetized with an intraperitoneal injection of ketamine (50 mg/kg; KetaVed, Vedco Inc., St. Joseph, MO, USA) and dexmedetomidine (0.5 mg/kg; Dexdomitor®, Orion Pharma, Espoo, Finland) in a 2:3 ratio. The mid–lower back area was shaved and cleaned with Nolvasan® wound cleaner (Zoetis, Parsippany, NJ, USA). Refresh® Lacrilube® ointment (AbbVie Inc., Chicago, IL, USA) was applied to the eyes to prevent dryness. The level of anesthesia was confirmed by the absence of bilateral corneal reflex and paw withdrawal reflex before surgery was started.

After surgery, the wound site was rinsed with 0.1% gentamycin in PBS before closing the muscles with 4-0 vicryl suture (Ethicon, Somerville, NJ, USA). The skin was closed with Michel wound clips (Fine Science Tools, Foster City, CA, USA) and then cleaned with hydrogen peroxide. Rats received a subcutaneous injection of Antisedan® (0.06 mL, 1.5 mg/mL; Orion Pharma, Espoo, Finland) for the reversal of the anesthesia, lactated Ringer’s (6 mL, 0.1 M, pH 7.4; Hospira Inc., Lake Forest, IL, USA) to restore fluids and electrolytes, gentamycin (0.03 mL, 6 mg/kg; GentaMax® 100, Clipper Distribution, St. Joseph, MO, USA) to prevent infections, and slow release buprenorphine (0.08 mL, 1.2 mg/kg; Buprenorphine SR, ZooPharm, Swedesboro, NJ, USA) for analgesia. Animals were monitored until awake and mobile. Gentamycin (0.03 mL, 6 mg/kg) was administered daily for 7 days after surgery and lactated Ringer’s (6 mL) during the first 3 days after surgery.

### 2.3. Spinal Cord Contusion

The skin over the mid to low thoracic back was incised and the exposed back muscles separated to expose the thoracic (T) vertebrae. The T8 vertebra was located and a laminectomy was performed to expose the underlying T9 spinal cord. The area was rinsed with a bath of 2% lidocaine (20 mg/mL; Hospira Inc., Lake Forest, IL, USA) for additional local anesthesia. The rat was then immobilized using vertebrae clamps on T7 and T9 and the exposed spinal cord contused at 175 kDyne (Infinite Horizon IH-0400 impactor; Precision Systems and Instrumentation LLC, Versailles, KY, USA).

### 2.4. Cell Preparation

**Figure 1:**
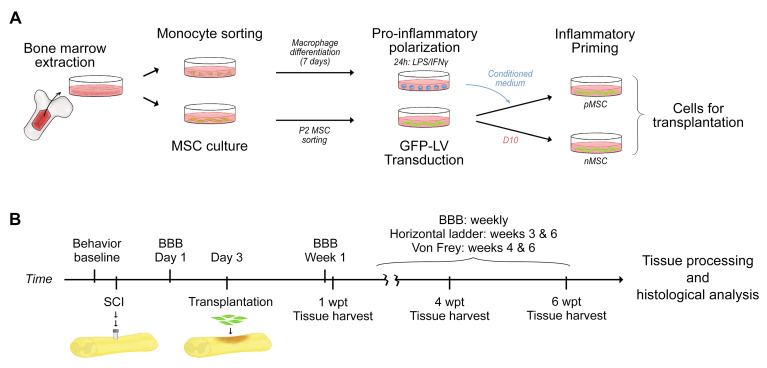
Experimental design. (**A**) The bone marrow was extracted from rats’ femurs and tibias and cultured on plastic dishes for 24 h. Then, monocytes were sorted using FACS and cultured for 7 days until differentiated to bone-marrow-derived macrophages. Mature macrophages were polarized to pro-inflammatory cells by culture in medium with LPS and IFNγ for 24 h. The conditioned medium (CM) from pro-inflammatory macrophages was collected and, later, used to prime MSC. In parallel, bone-marrow-derived MSC, adhered to the dish during the 24 h of bone marrow culture, were maintained in culture until passage 2, then FACS-sorted for purification. Sorted MSC were transduced with GFP-lentivirus to turn them green and trackable. P4 GFP-positive MSC were divided into two batches; one was cultured for 24 h in pro-inflammatory macrophage CM (primed MSC, pMSC) and the other one was cultured for 24 h in fresh D10 (naïve MSC, nMSC). pMSC and nMSC were trypsinized and resuspended in DMEM:F12 for transplantation. (**B**) Rats were acclimated to the behavioral tests before surgery, and the baseline scores for each test were obtained. Surgery for contusive SCI was performed. and the next day, BBB scores of the injured rats were collected. Three days after injury, surgery for direct injection of pMSC, nMSC, or DMEM:F12 was performed. At 1 and 4 wpt, six rats per group and time point were fixed and their spinal cords dissected for histological analysis. Ten additional rats per group were kept alive for 6 wpt, and their sensorimotor performance was measured using BBB scores weekly after transplant, the horizontal ladder test at 3 and 6 wpt, and the Von Frey test at 4 and 6 wpt. At 6 wpt, those rats were fixed and their spinal cords dissected for histological analysis. Abbreviations: MSC: mesenchymal stromal cell; P2: passage 2; LPS: lipopolysaccharide; IFNγ: interferon-gamma; GFP: green fluorescent protein; LV: lentivirus; D10: DMEM medium with 10% fetal bovine serum and 0.1% gentamycin; SCI: spinal cord injury; BBB: Basso, Beattie, Bresnahan locomotor scale; wpt: weeks post-transplant.

MSC and macrophages were obtained from bone marrow of the adult rat femurs and tibias following previously described procedures [27]. Briefly, bone marrow from femurs and tibias was flushed out with D10 medium (DMEM, ThermoFisher Scientific, Waltham, MA, USA; supplemented with 10% fetal bovine serum and 0.1% gentamycin) and cultured on plastic dishes in D10 at 37 °C and 6% CO_2_ for 24 h. Monocytes were sorted from the initial culture medium using fluorescence activated cell sorting (FACS) for the surface antigens CD45(+)/CD11b/c(+) [27]. MSC that attached to the plastic dish were further cultured for 3 days under the same conditions, harvested, and plated on plastic dishes coated with poly-D-lysine (PDL) in D10. These cells were passaged (P) twice to obtain P2 MSC (Figure 1).

The monocytes were cultured in RPMI-1640 (Roswell Park Memorial Institute medium; ThermoFisher Scientific, Waltham, MA, USA) supplemented with 10% fetal bovine serum, 0.1% gentamycin, and 50 ng/ml macrophage-colony stimulating factor (M-CSF, Peprotech, Rocky Hill, NJ, USA) to differentiate them into macrophages over 7 days. These macrophages were polarized to pro-inflammatory macrophages with 100 ng/mL lipopolysaccharide (LPS, Sigma-Aldrich, St. Louis, MO, USA) and 20 ng/ml interferon gamma (IFN-γ, Peprotech, Rocky Hill, NJ, USA). Twenty-four hours later, the supernatant of the inflammatory macrophages was collected, cleared of cell debris, and stored at −80 °C until used to prime MSC [27].

The P2 MSC were sorted using FACS for CD29(+)/CD45(−) [27], cultured in D10, and transduced with lentivirus to express green fluorescent protein (GFP). At P4, half of the transduced MSC were primed by culturing in inflammatory-macrophage-derived conditioned medium for 24 h (pMSC). The other half of the transduced MSC was cultured in fresh D10 (naïve MSC, nMSC). On the day of transplantation, GFP-positive pMSC or GFP-positive nMSC were trypsinized, counted, and resuspended in DMEM/F12 (Dulbecco’s Modified Eagle Medium: Nutrient Mixture F-12 (1:1); ThermoFisher, Waltham, MA, USA).

### 2.5. Cell Transplantation

Three days after contusion, rats were anesthetized, and the spinal cord exposed. Next, a total volume of 5 µL containing 1 × 10^6^ pMSC in DMEM/F12, 1 × 10^6^ nMSC in DMEM/F12, or DMEM/F12 only was injected in the injury epicenter at 1.5 mm depth into the cord and a rate of 1 µL/min using a 10 µL Hamilton syringe fitted with a pulled glass needle (100 µm internal diameter) and fixed to a stereotaxic device [16,29]. After injection, the needle was kept in place for 5 min to prevent backflow. Possible leakage was checked using a UV flashlight. The needle was gently withdrawn, the muscles were closed with 4-0 vicryl suture (Ethicon), the skin was closed with Michel wound clips (Fine Science Tools), and the skin surface cleaned with hydrogen peroxide. Acute transplantation (3 days after injury) was selected after a time course study showing best MSC transplant survival and repair outcome found in rat spinal cord after contusive SCI [16]. Rats survived for 1, 4, or 6 weeks post transplantation (wpt).

### 2.6. Tissue Harvest and Processing

Rats were euthanized by transcardial perfusion. Rats were given a lethal dose of ketamine and dexmedetomidine (as above), and their thorax was opened to expose the heart. The perfusion cannula was inserted through the left ventricle into the aorta and a small incision was made in the right atrium. The rats were perfused with 300 mL phosphate-buffered saline (PBS) until the blood was cleared, followed by 400 mL of 4% paraformaldehyde (PFA) in PBS (0.1 M, pH 7.4) until tissue fixation was achieved. The spinal cord was dissected out, kept in 4% PFA for 24 h at 4 °C, and then transferred to 30% sucrose in PBS (0.1 M, pH 7.4) for at least 48 h. Then, a 12 mm long segment centered at the injury epicenter was cut out, embedded in NEG-50 medium (ThermoFisher Scientific, Waltham, MA, USA), and frozen at –20 °C for approximately 2 h. The tissue was longitudinally sectioned on a Leica cryostat at 20 µm thickness. Sections were mounted in series on positively charged glass slides (VWR, Radnor, PA, USA) and stored at –20 °C until further use. 

### 2.7. Immunohistochemistry

Slides were thawed at room temperature and treated for 1 h with a blocking solution of PBS containing 5% normal goat serum (Vector Laboratories, Burlingame, CA, USA) to avoid non-specific binding. For staining of intracellular structures, the blocking solution also contained 0.3% Triton X-100 (Alfa Aesar, Ward Hill, MA, USA). Next, sections were incubated with the primary antibody (Table S1) in blocking solution for 2 h at room temperature, followed by overnight at 4 °C. The sections were washed three times for 5 min in PBS before incubation with the secondary antibody (Table S2) in PBS for 2 h at room temperature in the dark. Sections were washed three times for 5 min in PBS, stained with DAPI for 3 min, and washed twice for 5 min in PBS. Finally, the sections were air-dried and covered with glass slips (VWR, Radnor, PA, USA) using DAKO fluorescent mounting medium (Agilent Technologies, Santa Clara, CA, USA).

Sections were imaged using a VS120 Olympus slide scanner (Olympus Corporation, Tokyo, Japan). Overviews were taken at 4× magnification under bright field, and fluorescent images were taken at 20× magnification with exposures adjusted for each channel and staining. Autofocus parameters were collected from the DAPI channel. Image analysis of fluorescent images was performed with ImageJ.

### 2.8. MSC Transplant

**Figure 2:**
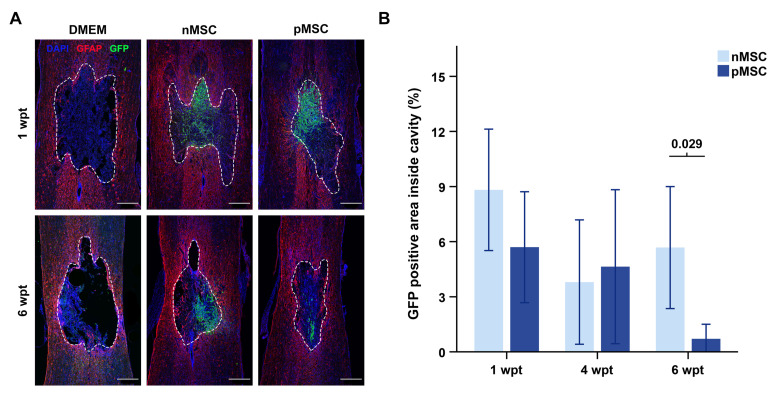
Inflammatory priming decreases long-term survival of MSC in the contused spinal cord. (**A**) Photomicrographs representing examples of injured spinal cords at 1 and 6 wpt for the vehicle injected animals (DMEM), the nMSC transplanted animals, and the pMSC transplanted animals. The immunostaining marks astrocytes in red (GFAP), transplants in green (GFP), and cell nuclei in blue (DAPI). The dashed lines represent the manually selected regions of interest (ROIs) to designate the injury cavity. Any green fluorescence detected on the DMEM images is a product of auto fluorescence, since those animals did not receive LV-GFP transduced cells. Scale bars represent 500 µm (Overview: 4×, image scan: 20×). The spinal cords’ orientation is top–rostral and bottom–caudal. Intensity has been equally modified on all images for illustrative purposes only but not for quantitative analysis. (**B**) Bar graph representing the percentage of the injury cavity occupied by GFP-positive area over time from those animals that received a cell transplant. pMSC occupy eight-fold less area than nMSC at 6 wpt. The DMEM group was not included in the graph because the values are negligible. The bars represent mean and SEM. *n* = 6 per group and time point. Statistical significance (noted on the graph) was accepted at *p* < 0.05. *Abbreviations*: DMEM: Dubbelco’s Modified Eagle’s Medium; nMSC: naïve MSC; pMSC: primed MSC; wpt: weeks post-transplant; GFP: green fluorescent protein; MSC: mesenchymal stromal cell; GFAP: glial fibrillary acidic protein.

Sections from rats at 1, 4, or 6 wpt were stained with antibodies against GFP and the astrocyte marker, glial fibrillary acidic protein (GFAP), and used to assess the MSC transplant in the contused spinal cord segment. The GFAP-staining was used to delineate the regions of interest (ROI; Figure 2). The GFP-positive area was determined within and outside of the ROI after subtracting the background using the Gaussian blur. Results were presented as the percentage of the total area inside the injury. Values from the three middle sections per animal were summed, and the mean per group and time point were calculated.

### 2.9. Spinal Cord Nervous Tissue

**Figure 3:**
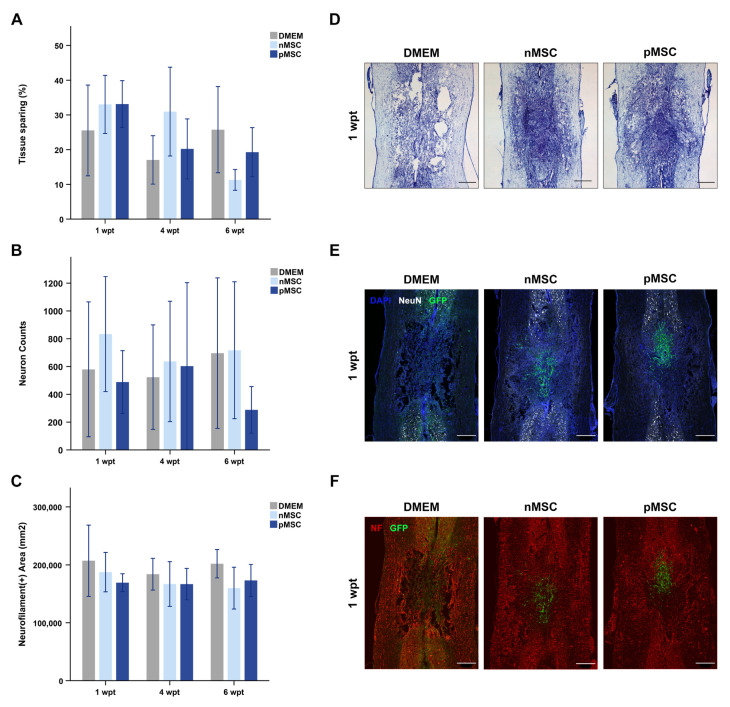
All experimental groups have similar effects on nervous tissue sparing. (**A**) Bar graph representing the percentage of healthy tissue spared, relative to an equally sized healthy spinal cord segment over time. No significant differences were found between groups. (**B**) Bar graph representing the number of neurons present just rostral and caudal to the injury over time. No significant differences were found between groups. (**C**) Bar graph representing axonal presence in the injury over time. No significant differences were found between groups. On (**A**–**C**), bars represent the mean and SEM. *n* = 6 per group and time point. (**D**–**F**) Example photomicrographs of injured spinal cords at 1 wpt are shown for visualization purposes. The spinal cords’ orientation is top–rostral and bottom–caudal. (**D)** Tissue sparing was quantified using cresyl violet staining imaged under a bright field microscope (2.5× magnification). (**E**) Neuronal bodies were quantified using NeuN (white), GFP (green), and DAPI (blue) staining. (**F**) Axonal presence was quantified using NF (red) and GFP (green) staining. For (**E**,**F**), intensity was equally modified on all images for illustrative purposes only but not for quantitative analysis (Overview: 4×, image scan: 20×). Any green fluorescence detected on the DMEM images on E and F is product of auto fluorescence since those animals did not receive LV-GFP transduced cells. Scale bars on all images represent 500 µm. Abbreviations: DMEM: Dubbelco’s Modified Eagle’s Medium; nMSC: naïve MSC; pMSC: primed MSC; wpt: weeks post-transplant; GFP: green fluorescent protein; MSC: mesenchymal stromal cell; NeuN: neuronal nuclei; NF: neurofilament.

One series of sections was stained with cresyl violet. Cresyl-violet-stained sections from rats at 1, 4, or 6 wpt were used to assess the volume of healthy nervous tissue remaining in the contused spinal cord segment using an Axio Observer Z1 inverted microscope (Zeiss, Thornwood, NY, USA). The volume of spared nervous tissue was quantified using the Cavalieri estimator function on the StereoInvestigator® stereology software (MBF Bioscience, Williston, VT, USA) as previously described ([6]; Figure 3A). Spared tissue volumes were expressed as a percentage of the volume of an equally sized segment of an uninjured spinal cord taken from rats of similar age and similarly processed. The mean per group and time point was calculated.

We determined neuron presence on sections stained with antibodies against neuronal nuclei (NeuN; Figure 3B) in two ROIs (each 1.5 mm × 1 mm) just rostral and caudal to the injury. We created a mask of NeuN staining, quantified DAPI particles within the NeuN mask, and normalized the counts to the average DAPI particle size to correct for overlapping or truncated nuclei. The values were summed per section and per rat. We used sections stained with antibodies against neurofilament (NF) to assess axon presence in the contused spinal cord segment (Figure 3C) in an ROI (1.5 mm × 2.5 mm) centered at the epicenter. The values obtained per section of the same animal were averaged. Values for NeuN and NF were acquired after background correction using the Gaussian blur with a sigma value chosen for each staining. Outcome measures were averaged per group and time point.

### 2.10. Assessment of Macrophage Phenotype

**Figure 4:**
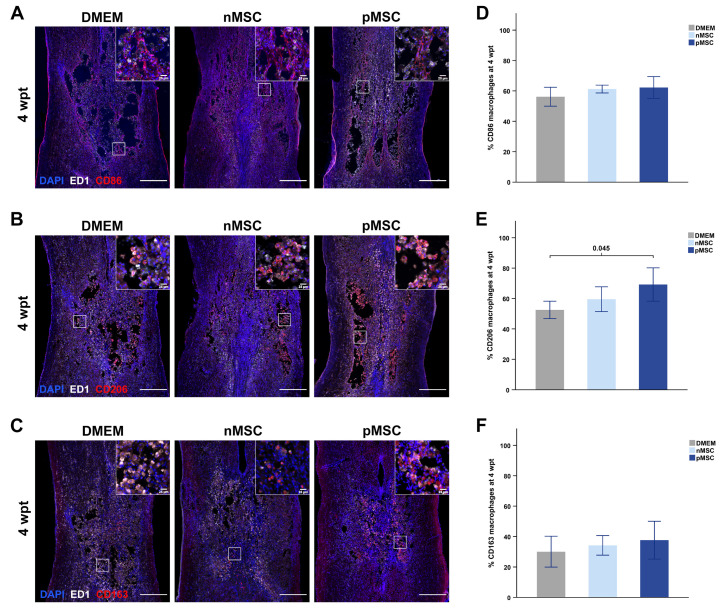
pMSC transplants induce transient immunomodulation at 4 wpt. (**A**–**C**) Example photomicrographs of injured spinal cords at 4 wpt. Inlets represent a close-up detail of the staining; a square on the overview designates the source for the inlet image. The spinal cords’ orientation is top–rostral and bottom–caudal. (**A**) Pro-inflammatory macrophages were quantified using CD86 (red), ED1 (white), and DAPI (blue) staining. (**B**) Early-acting pro-inflammatory macrophages were quantified using CD206 (red), ED1 (white), and DAPI (blue) staining. (**C**) Late-acting pro-inflammatory macrophages were quantified using CD163 (red), ED1 (white), and DAPI (blue) staining. For (**A**–**C**)**,** intensity was equally modified on all images for illustrative purposes only but not for quantitative analysis. Scale bars on all images represent 500 µm for the overviews and 25 µm for the inlets (Overviews: 4×, image scan: 20×, Inlets: 20×). (**D**) Bar graph representing the percentage of pro-inflammatory macrophages, relative to the total number of macrophages in the injury at 4 wpt. No significant differences were found between groups. (**E**) Bar graph representing the percentage of early anti-inflammatory macrophages, relative to the total number of macrophages in the injury at 4 wpt. pMSC transplants result in 20% more CD206-positive macrophages compared to controls. (**F**) Bar graph representing the percentage of late anti-inflammatory macrophages, relative to the total number of macrophages in the injury at 4 wpt. No significant differences were found between groups. On (**D**–**F**), bars represent the mean and SEM, and significance was accepted at *p* < 0.05. *n* = 6 per group and time point. Abbreviations: DMEM: Dubbelco’s Modified Eagle’s Medium; nMSC: naïve MSC; pMSC: primed MSC; wpt: weeks post-transplant; MSC: mesenchymal stromal cell.

We used sections stained with antibodies against the cell surface marker CD86 (pro-inflammatory), CD206 (early anti-inflammatory), and CD163 (late anti-inflammatory) to assess the presence of macrophages in different polarization states (Figure 4). The distinction between early and late anti-inflammatory macrophages refers to their sequential function in the modulation (CD206) and resolution (CD163) of the inflammatory response [30,31]. Sections were also stained with antibodies against ED1 (CD68) to assess the total presence of macrophages in the contused spinal cord segment. ROIs (1.5 mm × 3 mm) were centered at the injury epicenter. The background was removed using Gaussian blurs with a channel-specific sigma. A double positive area mask for ED1 and each macrophage marker was created and overlaid on the DAPI channel to quantify double positive cells for each of the polarization states. We quantified ED1-positive macrophages using a single mask over the DAPI channel. The Watershed plug-in was used on the DAPI image before quantification to facilitate counts in high cell density areas. We performed DAPI particle size standardization on the final counts to correct for overlapping or truncated nuclei. The counts obtained per section were averaged for the same animal and averaged per group and time point.

### 2.11. Assessment of Blood Vessels

**Figure 5:**
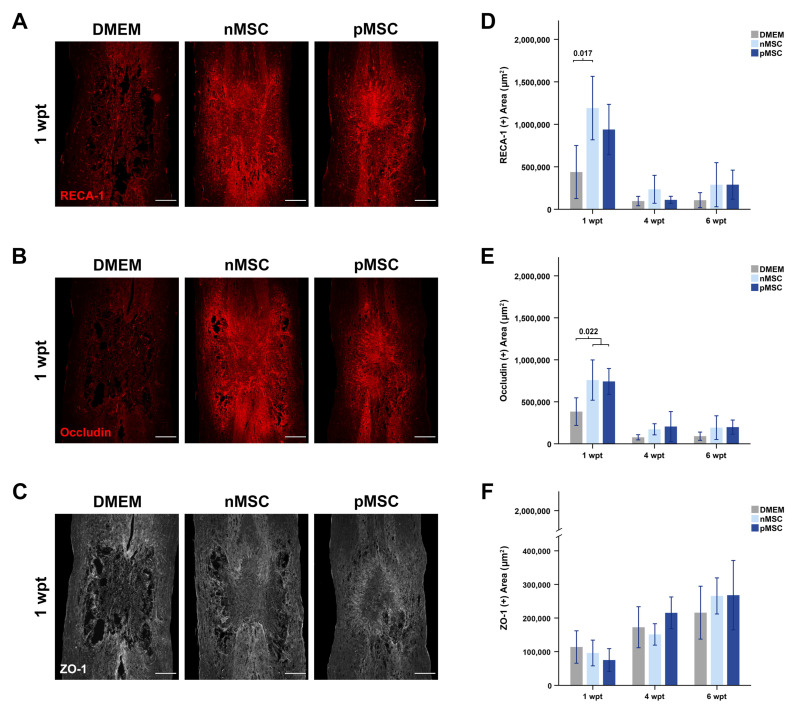
pMSC and nMSC transplants promote angiogenesis at 1 wpt. (**A**–**C**) Example photomicrographs of injured spinal cords at 1 wpt. The spinal cords’ orientation is top–rostral and bottom–caudal. (**A**) Blood vessel presence in the injury was measured using RECA-1 (red) staining. (**B**) Blood vessel maturation in the injury was measured using occludin (red) staining, and (**C**). ZO-1 (white) staining. For (**A**–**C**), intensity was equally modified on all images for illustrative purposes only but not for quantitative analysis. Scale bars on all images represent 500 µm (Overviews: 4×, image scan: 20×). (**D**) Bar graph representing the area of RECA-1 in the epicenter of the injury over time. nMSC transplants result in almost three-fold more blood vessel presence compared to controls at 1 wpt. (**E**) Bar graph representing the area of occludin in the injury epicenter over time. nMSC and pMSC transplants result in two-fold more occludin presence compared to controls at 1 wpt. (**F**) Bar graph representing the area of ZO-1 in the injury epicenter at 1 wpt. No significant differences were found between groups. In (**D**–**F**), bars represent the mean and SEM, and significance was accepted at *p* < 0.05. *n* = 6 per group and time point. Abbreviations: DMEM: Dubbelco’s Modified Eagle’s Medium; nMSC: naïve MSC; pMSC: primed MSC; wpt: weeks post-transplant; MSC: mesenchymal stromal cell; RECA-1: rat endothelial cell antigen 1; ZO-1: zonula occludens 1.

We used sections stained for the rat endothelial cell antigen (RECA-1) to assess blood vessel presence (Figure 5). Blood vessel maturation was assessed on sections stained with antibodies against occludin and zonula occludens-1 (ZO-1). After background removal using the Gaussian blur for each staining, areas positive for RECA-1, occludin, and ZO-1 were measured in ROIs (1.5 mm × 2 mm) centered at the injury epicenter. The values obtained from the three central sections on the slide, representing 540 µm of the injury, were averaged per animal, and the mean per group and time point was calculated.

### 2.12. Assessment of Hind Limb Sensorimotor Performance

Hind limb locomotor performance was evaluated using the BBB open field locomotor test [32,33]. Rats were allowed to acclimate to the open field environment and were tested by two independent testers blinded to the treatments. Rats were tested before the surgery (i.e., baseline scores), one day after injury, and once weekly after transplantation. Sensorimotor coordination was assessed using the horizontal ladder test [34] before surgery, for acclimation and baseline calculation, at 3 weeks, and 6 weeks after transplantation. We quantified the total number of steps and the number of small, medium, and large slips of the foot on the horizontal ladder. We presented the total number of slips as a percentage of the total steps. Sensory function was evaluated by measuring the level of mechanical allodynia using the Von Frey test [35]. Rats were acclimated to the test environment before measurements. The Von Frey filaments were applied perpendicular to the mid-plantar area of each hind paw, the pressure was increased until paw withdrawal, and the grams (g) of force applied at withdrawal were recorded. The measurements of both paws were averaged. Measurements were obtained before contusion and at 4 and 6 weeks after transplantation. The scores for each behavioral test were averaged per experimental group and analyzed over time.

### 2.13. Statistical Analysis

This study was conducted with a completely randomized design. The number of animals chosen to achieve statistical power was based on literature research and pilot experiments. Statistical analyses were performed with the IBM SPSS Statistics software 26 (IBM, Armonk, NY, USA). Data were tested for normality before determining the appropriate statistical test. For histological outcomes, the means per group and time point were compared and tested for statistical significance, which was accepted with *p* ≤ 0.05. For parametric data with two or three experimental groups, student’s *t*-test or one-way analysis of variance (ANOVA) were used, respectively, to compare means per group within each time point. Kruskall–Wallis test was used if data from three experimental groups were determined non-parametric. Bonferroni correction was used for multiple comparisons after ANOVA or Kruskall–Wallis tests. Correlation analysis was performed to evaluate the association between transplant survival and therapeutic outcomes, and analysis of covariance was performed to study the therapeutic outcomes between groups when controlling for transplant survival. Behavioral data were analyzed using repeated measures ANOVA to compare means across time points.

## 3. Results

### 3.1. Study Design

Animals were randomly divided into three groups across three time points. We compared the effect of a pMSC transplant with that of an nMSC transplant or DMEM injection (k = 3) in a contusion model of SCI (Figure 1). Rats fixed at 1 wpt (*n* = 6 per group) or 4 wpt (*n* = 6 per group) were used for histological analysis. Rats fixed at 6 wpt (*n* = 10 per group) were used for histological and behavioral analysis. From the 6 wpt cohort, six rats per group were used for histological analysis (*n* = 6 per group). The spinal cords of all rats were dissected and processed for histology to evaluate the biological effect of inflammatory priming on the MSC transplant and the spinal cord nervous tissue. Sensorimotor skills of all rats in the 6 wpt cohort were tested weekly throughout the experiment. Ninety percent survival was achieved throughout the experiment. Deceased rats were replaced to obtain the group size described above. 

### 3.2. MSC Transplant Survival

The area of GFP-positive MSC was determined in the injury, which was delineated inside GFAP-positive staining (Figure 2A). The percentage of the cavity area occupied by nMSC (M = 8.82, SEM = 1.51) and pMSC (M = 5.7, SEM = 1.65) was similar at 1 wpt (t(10) = –1.39, *p* = 0.194) and 4 wpt (nMSC; M = 3.8, SEM = 1.69; pMSC; M = 4.64, SEM = 2.1; (t(10) = 0.31, *p* = 0.762) (Figure 2B). However, at 6 wpt, the percentage of cavity area occupied by pMSC (M = 0.71, SEM = 0.34) was eight-fold smaller (t(5.56) = −2.91, *p* = 0.029) than that of nMSC (M = 5.68, SEM = 1.66) (Figure 2B). These data revealed that inflammatory priming reduced long-term survival of MSC in a spinal cord contusion environment.

### 3.3. Effects of pMSC on the Contused Spinal Cord

The volume of spared nervous tissue (Figure 3A), neuron presence (Figure 3B), and axon presence (Figure 3C) in the injured spinal cord segment were calculated to assess neuroprotection. The effect of pMSC, nMSC transplants and controls was similar on all three outcomes at all the examined time points after transplantation. All data are summarized in Table 1. Priming MSC did not improve the neuroprotective ability of the transplant in this SCI model.

The macrophage phenotype was studied to assess immunomodulation following MSC transplantation. Pro-inflammatory macrophages were recognized by the presence of CD68 (ED1) and CD86 (Figure 4A). We used the presence of CD206 to recognize early acting anti-inflammatory macrophages (Figure 4B) and CD163 for late acting anti-inflammatory macrophages (Figure 4C). At 4 wpt, we found that rats with a pMSC transplant had a significant increase of about 20% in early anti-inflammatory macrophages, relative to total macrophage count, compared with control rats (F(2,15) = 3.841, *p* = 0.045) (Figure 4E). At this time point, there were no differences in the relative amounts of pro-inflammatory (Figure 4D) and late acting anti-inflammatory (Figure 4F) macrophages. There were no statistically significant differences in macrophage populations between the experimental groups at 1 or 6 wpt. The results are summarized in Table 1 and shown in supplementary data (Figure S1).

The presence of blood vessels was examined using RECA-1 immunostaining (Figure 5A), and blood vessel maturation was assessed using occludin (Figure 5B) and ZO-1 immunostaining (Figure 5C). At 1 wpt, we found significantly more blood vessels in the injury epicenter in rats with nMSC compared with controls (F(2,15) = 5.419, *p* = 0.017) (Table 1, Figure 5D). In rats with nMSC or pMSC, we found significantly higher occludin presence compared with controls (F(2,15) = 5.009, *p* = 0.022) (Table 1, Figure 5E). There were no statistically significant differences between groups at 4 wpt (Figure 5D) and 6 wpt (Figure 5E) for RECA-1 or occludin area. We found no differences in ZO-1 presence between experimental groups at any of the time points examined (Figure 5F). We further analyzed RECA-1 presence just rostral, central, and caudal to the injury (Figure S2). In rats with a pMSC transplant, significantly more blood vessels were found in the rostral region at 4 wpt compared with rats with an nMSC transplant or control rats (Figure S2A).

### 3.4. Therapeutic Outcomes Associated with Transplant Presence

To determine the influence of the limited transplant survival on the therapeutic potential of pMSC, we performed correlation and covariance analyses. We correlated each therapeutic outcome with transplant presence (Table 1, last column), and we used transplant presence as a covariate for ANCOVA. Our data revealed a moderately strong correlation between the presence of pMSC and the volume of nervous tissue spared (Table 1). We also found a significant and moderately strong correlation between the amount of nMSC and the presence of early anti-inflammatory macrophages and an inverse strong correlation between nMSC presence and late anti-inflammatory macrophages (Table 1). Finally, a strong correlation was found between nMSC or pMSC transplant presence and RECA-1 and occludin areas (Table 1). A moderately strong inverse correlation was found between pMSC transplant presence and ZO-1 area (Table 1).

Our analysis of covariance revealed a significant effect of MSC transplant presence on macrophage phenotype. After correcting our model for the amount of surviving transplant, pMSC transplants were associated with significantly higher percentages of pro-inflammatory (F(1,33) = 6.702, *p* = 0.014) and early anti-inflammatory (F(1,33) = 5.923, *p* = 0.021) macrophages across time points. This effect was not found for nMSC transplants or for late anti-inflammatory macrophages. Together, our data suggest that sub-acute transplantation of inflammation primed MSC modulates the local inflammatory microenvironment at the later stages following SCI and transplantation. These data also suggest that successful healthy tissue sparing and revascularization are affected by the amount of pMSC transplant present at the injury site over time.

### 3.5. Hind Limb Sensorimotor Performance was Unchanged

We evaluated the effect of pMSC transplants on locomotor and sensory recovery after spinal cord contusion. In the over-ground walking test, all rats had a BBB score of 21 before surgery and dropped below 5 after contusion. We found a significant increase in locomotor performance of the vehicle control injected group compared to the nMSC and pMSC injected groups at 1 wpt, but the effect was not sustained beyond that time point. Thereafter, the scores were similar between groups and did not increase over time (Figure S3A). In addition, we found no significant differences between groups on the percentage of slips occurred on the horizontal ladder test (Figure S3B) at 3 or 6 wpt or in the magnitude of mechanical allodynia obtained by the Von Frey test (Figure S3C) at 4 or 6 wpt. Overall, these data indicate that inflammatory priming of MSC did not improve their therapeutic effect on functional recovery.

## 4. Discussion

Macrophage-derived inflammatory priming of MSC leads to upregulation of genes and secreted proteins that support tissue repair and downregulation of cell survival genes [27]. Here, we showed that transplanted pMSC exhibit reduced long-term survival in an adult rat spinal cord contusion. We showed that pMSC transplants induce transient immunomodulatory effects on the local macrophages. We found that the magnitude of tissue sparing, blood vessels, and occludin found at the injury site increased proportionally to the magnitude of pMSC transplant survival. Our data indicate that inflammatory priming increased the immunomodulatory potential of MSC but did not enhance the neuroprotective, angiogenic, or functional recovery effects of pMSC on the injured spinal cord beyond those elicited by nMSC. Here, we discuss how the reduced transplant survival could be limiting the therapeutic effect of pMSC transplants.

### 4.1. Inflammatory Priming and Transplant Survival in SCI

Inflammatory priming shortened the survival of MSC following transplantation into a spinal cord contusion. Relative to nMSC transplants, survival of pMSC transplants was significantly lower at 6 weeks after transplantation. A spinal cord contusion is a hostile environment for MSC transplants due to cellular debris, a long lasting pro-inflammatory immune response, and free radicals [36,37,38], all of which can induce stress pathways in MSC [28] that can trigger senescence or apoptosis [39,40]. Macrophage-derived inflammation priming of MSC prior to transplantation activates such pathways [27], which sensitizes MSC to the cellular hostility in the contusion environment, thereby, reducing the transplant’s lifetime. Previous research has shown that pre-activation of MSC with inflammatory stimuli increases their sensitivity to respond to a subsequent inflammatory stimulus [41]. Interferon-gamma (IFNγ) and tumor necrosis alpha (TNFα) are secreted by pro-inflammatory macrophages and are known stimulants of apoptotic pathways as a physiological mechanism in response to infection or injury [42,43,44]. The presence of these two factors in the macrophage-derived priming medium could be responsible, at least in part, for the reduced pMSC transplant survival in the contused spinal cord.

### 4.2. The primed Secretome and Nervous Tissue Sparing

Inflammation priming of MSC increases their secretion of neurotrophic growth factor (NGF) and glial derived neurotrophic factor (GDNF) [27], both of which support neuronal survival and neurite outgrowth in vitro and in vivo [45,46]. In models of SCI, treatment with NGF and BDNF results in reduced injury size, axon sprouting, neuronal survival, and functional recovery [47,48,49]. In the present study, significant improvements in neuroprotection or functional recovery were not observed following transplantation of pMSC compared with nMSC or the vehicle control. However, we found a significant moderate correlation between the amount of pMSC present in the injury and the amount of spared tissue. These results indicate that neuroprotective actions by inflammation primed MSC could be curbed by reduced transplant survival.

### 4.3. Immunomodulation by Primed MSC

Interactions between MSC and immune cells are necessary to activate MSC’s immunomodulatory activity when participating in tissue repair [19,50]. Macrophage-derived priming of MSC induces upregulation of immunomodulatory genes, such as indoleamine 2,3-dioxygenase [27]. In the current study, we show that pMSC transplantation resulted in higher percentage of anti-inflammatory CD206-positive macrophages at the injury site at 4 weeks after transplantation. CD206-expressing macrophages are involved in the resolution of inflammation [31]. We also observed a significant increase in both CD86-positive and CD206-positive macrophages with the pMSC transplant compared with the nMSC transplant after accounting for the variability of transplant survival. These outcomes indicate that, while the immunomodulatory ability of pMSC is limited by transplant survival, the priming strategy transiently enhances the anti-inflammatory effect of MSC. Toll like receptor-4 (TLR4) activation by ligands, such as lipopolysaccharide (LPS) or damage-associated high mobility group box 1 (HMGB-1), modifies MSC behavior, inducing their immunosuppressive abilities [51,52,53,54]. It is possible that the residual presence of LPS in the priming medium, and HMGB1 in the injury [55], activated TLR4 and enhanced the immunomodulatory actions by pMSC. Chronic inflammation in SCI contributes to secondary injury and limited repair [14,38,56]; therefore, modification of the local inflammatory environment can elicit neuroprotection and functional recovery [7,57,58]. Interestingly, previous reports show that apoptotic MSC have stronger immunomodulatory effects than live MSC on neighboring macrophages, as evidenced by upregulated prostaglandin E2 (PGE2) in macrophages after transplantation of apoptotic MSC [59,60]. Those findings allude to a sacrificial role of MSC that, in response to inflammatory stimuli, contributes to the resolution of inflammation.

### 4.4. The Primed Secretome and Revascularization after SCI

Priming with inflammatory stimuli also triggers upregulation of growth factors that are key in the generation and maturation of blood vessels, such as vascular endothelial growth factor (VEGF), hepatocyte growth factor (HGF), angiopoietins, and endothelial growth factor (EGF) [27,28,61]. Administration of VEGF and platelet derived growth factor (PDGF) to an SCI causes revascularization and neural repair [62]. In our model of SCI, both pMSC and nMSC elicited increased occludin presence to a similar extent in the injury epicenter one week after transplantation. However, we found strong correlations between the level of revascularization (based on RECA-1 and occludin presence) and the amount of MSC survival in the injury site. Considering that the observed revascularization level was the same for nMSC and pMSC at six weeks after injury, while the amount of transplant was significantly reduced for pMSC, it may be that priming induced long-lasting upregulation of angiogenic factors. These outcomes support the concept that therapeutic effects of pMSC are limited by the reduced transplant survival. We also observed that the two tight junction proteins, occludin and ZO-1, which are key in the maturation and sealing of newly generated blood vessels [63,64,65], covered the same amount of area as RECA-1 staining at six weeks after transplantation. This suggests that both nMSC and pMSC support blood vessel maturation and functionalization.

### 4.5. MSC Priming

Enhancing MSC molecular predisposition to elicit nervous tissue repair is a promising strategy for treating SCI. MSC naturally home to damaged tissues following a gradient that modifies their phenotype, preparing them to efficiently interact with the injury environment and support repair [66,67]. However, MSC do not naturally home to the damaged spinal cord. Typically, untreated MSC are transplanted in the spinal cord, which could cause sudden environmental shock for the cells. Other MSC priming methods (i.e., cytokines, hypoxia, antioxidants, chemical drugs) have been used to increase their therapeutic efficacy in a variety of tissues, resulting in enhanced repair [20,68,69,70,71]. However, the effect of those priming methods on transplant survival are varied or not always presented. A culture of MSC with IFNγ and TNFα added to the medium increases MSC immunomodulatory activity [21,23,68,72]. Pre-treatment of MSC with antioxidants, such as melatonin or all-trans retinoic acid, results in upregulation of the survival gene, bcl2, and increased MSC proliferation and wound healing efficacy, respectively [69,70]. The secretome of macrophages in different polarization states could also affect MSC survival and repair potential differently [73], which hints at the coordinated action between MSC and macrophages during wound healing [74]. This body of data indicates that further understanding of the mechanisms underlying the interactions between MSC and their environment will help to optimize the current priming method and better balance the transplant’s homeostasis and survival, and therapeutic efficacy for spinal cord repair. The ability to fine-tune MSC’s therapeutic output with different priming methods would be of special interest to the various models of SCI, characterized by different levels of pathology, such as the contusion and transection injuries [75].

### 4.6. Tackling Long Term Transplant Survival

Another possible explanation to the phenomena observed in this study could be that inflammatory priming and subsequent exposure to the injury environment involve the continued activation of cellular stress pathways, which could lead to increased cellular senescence [39]. MSC have intrinsic mechanisms to maintain homeostasis or trigger cell death when homeostasis is not sustained [39]. If cellular senescence is reached, pMSC may be self-activating apoptotic pathways in addition to the external triggers. A potential strategy to interfere in this behavior is to promote autophagy in the priming phase, which has been shown to be a homeostasis preservation mechanism in MSC that, when enhanced, results in better transplant health and survival [40].

## 5. Conclusions

Our study shows that macrophage-derived inflammatory priming of MSC before transplantation into SCI shortens the lifetime of the transplant but temporarily increases its anti-inflammatory effects, which could potentially be beneficial for SCI repair [7,57]. Also, we show the association between the magnitude of some of the therapeutic outcomes measured and the magnitude of transplant survival in the injury site, both for pMSC and nMSC transplants. Research is ongoing in our group to further elucidate the pathways involved in this priming effect, to characterize and isolate the components of the priming medium that induce the various effects seen on pMSC, and to optimize the priming method to allow for both improvement of the therapeutic potential and MSC survival.

## Figures and Tables

**Table 1:** Summary of therapeutic outcomes for nervous tissue repair.

Repair Effect	Outcome	Group	1 wpt	4 wpt	6 wpt	Correlation ^†^
Neuroprotection	Tissue sparing ^a^ (%)	pMSC	33.1 ± 3.4	20.2 ± 4.3	19.3 ± 3.5	0.592 **
		nMSC	33.0 ± 4.2	30.9 ± 6.4	11.3 ± 1.5	0.051
		DMEM	25.5 ± 6.5	17.0 ± 3.5	25.8 ± 6.2	N/A
	Neuronal survival ^b^ (counts)	pMSC	489 ± 113	603 ± 301	288 ± 84	0.398
		nMSC	834 ± 207	637 ± 217	717 ± 247	0.266
		DMEM	580 ± 243	524 ± 188	696 ± 271	N/A
	Axonal presence ^c^ (µm^2^)	pMSC	169,261 ± 7684	166,872 ± 13,592	173,308 ± 13,812	−0.255
		nMSC	187,484 ± 17,010	167,010 ± 19,376	159,952 ± 18,065	0.355
		DMEM	207,198 ± 30,776	183,902 ± 13,679	202,085 ± 12,265	N/A
Immunomodulation	Pro-inflammatory ^d^ (%)	pMSC	82.3 ± 2.8	62.2 ± 3.6	62.8 ± 3.4	0.340
		nMSC	68.3 ± 6.4	61.2 ± 1.3	54.2 ± 3.0	0.425
		DMEM	82.3 ± 7.9	56.2 ± 3.1	69.3 ± 5.5	N/A
	Early anti-inflammatory ^e^ (%)	pMSC	91.3 ± 6.1	69.2 ± 5.5 *	63.9 ± 5.1	0.298
		nMSC	75.8 ± 12.1	59.5 ± 4.1	57.5 ± 2.5	0.573 *
		DMEM	92.9 ± 6.0	52.5 ± 2.9	65.3 ± 2.2	N/A
	Late anti-inflammatory ^f^ (%)	pMSC	14.51 ± 2.5	37.6 ± 6.2	34.99 ± 2.5	−0.237
		nMSC	13.46 ± 2.7	30.06 ± 5.1	40.09 ± 5.5	−0.550 *
		DMEM	18.08 ± 4.2	34.2 ± 3.2	29.6 ± 5.2	N/A
Revascularization	Blood vessels ^g^ (µm^2^)	pMSC	938,637 ± 148,407	110,756 ± 21,157	289,606 ± 85,683	0.614 **
		nMSC	1,191,049 ± 186,800 *	235,060 ± 82,194	288,927 ± 129,815	0.645 **
		DMEM	438,311 ± 155,990	96,567 ± 27,864	107,048 ± 44,112	N/A
	Occludin ^h^ (µm^2^)	pMSC	742,582 ± 76,849 *	205,501 ± 89,115	198,592 ± 42,185	0.606 **
		nMSC	758,800 ± 119,874 *	172,563 ± 33,095	192,260 ± 70,535	0.568 *
		DMEM	383,145 ± 82,063	771,11 ± 15,488	90,500 ± 24,577	N/A
	ZO-1 ^i^ (µm^2^)	pMSC	74,904 ± 16,987	215,440 ± 23,596	267,937 ± 516,33	−0.595 **
		nMSC	95,962 ± 19,165	151,201 ± 15,934	265,711 ± 26,818	−0.304
		DMEM	113,779 ± 24,084	172,522 ± 30,525	215,962 ± 39,358	N/A

Table legend: Histological outcome values represent the mean and the standard error of the mean (SEM). ^a^ Percentage of tissue sparing in the contused spinal cord in reference to an equally sized segment of a healthy cord; ^b^ sum of neuronal bodies rostral and caudal to the injury cavity; ^c^ area of neurofilament positive staining representing axonal presence in the contused spinal cord; ^d^ percentage of macrophages positively stained for CD86 in the injury site; ^e^ percentage of macrophages positively stained for CD206 in the injury site; ^f^ percentage of macrophages positively stained for CD163 in the injury site; ^g^ area of RECA-1 positive staining in the injury site; ^h^ Area of occludin-positive staining in the injury site; ^i^ Area of ZO-1 positive staining in the injury site. (*) One asterisk indicates statistical significance when *p* ≤ 0.05, (**) and two asterisks indicate statistical significance when *p* ≤ 0.01. (†) The right-most column presents the Pearson correlation coefficient from evaluating the correlation between the histological outcome on each row and the amount of transplant present in the injury site. The correlation coefficient has an asterisk when found statistically significant. Abbreviations: pMSC: inflammation primed MSC, nMSC: naïve MSC, DMEM: injection control of DMEM/F12, wpt: weeks post-transplant, N/A: not applicable.

## Data Availability

The data presented in this study are available on request from the corresponding author.

## Supplementary Materials

The following are available online at https://www.mdpi.com/article/10.3390/cells10061316/s1. Table S1: Primary antibodies used in this study, Table S2: Secondary antibodies used in this study, Figure S1: Macrophage inflammatory phenotype over time, Figure S2: Blood vessel presence in the rostral, central, and caudal regions of the injury, Figure S3: Hind limb sensorimotor performance.

## Abbreviations

Bcl2	B-cell lymphoma 2 gene
DAPI	4′,6-diamidino-2-phenylindole
DMEM	Dubbelco’s Modified Eagle Medium
EGF	epidermal growth factor
FACS	fluorescence-activated cell sorting
GDNF	glial derived neurotrophic factor
GFAP	glial fibrillary acidic factor
GF	growth factors
HGF	hepatocyte growth factor
IACUC	Institutional Animal Care and Use Committee
IFNγ	interferon gamma
LPS	lipopolysaccharide
NeuN	neuronal nuclei
NF	neurofilament
NGF	nerve growth factor
nMSC	naïve MSC
P2	passage 2
PBS	phosphate-buffered saline
PDL	poly-D-lysine
PGE2	prostaglandin-E2
pMSC	primed MSC
RECA-1	rat endothelial cell antigen 1
ROI(s)	region(s) of interest
SCI	spinal cord injury
SR	slow release
T8	thoracic vertebra/segment 8
TNFα	tumor necrosis factor alpha
VEGF	vascular endothelial growth factor
wpt	weeks post-transplant
ZO-1	zonula occludens 1

## References

[1] Drago D., Cossetti C., Iraci N., Gaude E., Musco G., Bachi A., Pluchino S. (2013). The stem cell secretome and its role in brain repair. Biochimie.

[2] Johnson T.V., DeKorver N.W., Levasseur V.A., Osborne A., Tassoni A., Lorber B., Heller J.P., Villasmil R., Bull N.D., Martin K.R. (2014). Identification of retinal ganglion cell neuroprotection conferred by platelet-derived growth factor through analysis of the mesenchymal stem cell secretome. Brain.

[3] Asami T., Ishii M., Fujii H., Namkoong H., Tasaka S., Matsushita K., Ishii K., Yagi K., Fujiwara H., Funatsu Y. (2013). Modulation of murine macrophage TLR7/8-mediated cytokine expression by mesenchymal stem cell-conditioned medium. Mediat. Inflamm..

[4] Sun G., Li G., Li D., Huang W., Zhang R., Zhang H., Duan Y., Wang B. (2018). hucMSC derived exosomes promote functional recovery in spinal cord injury mice via attenuating inflammation. Mater. Sci. Eng. C Mater. Biol. Appl..

[5] Liu Y., Dulchavsky D.S., Gao X., Kwon D., Chopp M., Dulchavsky S., Gautam S.C. (2006). Wound repair by bone marrow stromal cells through growth factor production. J. Surg. Res..

[6] Ritfeld G.J., Rauck B.M., Novosat T.L., Park D., Patel P., Roos R.A., Wang Y., Oudega M. (2014). The effect of a polyurethane-based reverse thermal gel on bone marrow stromal cell transplant survival and spinal cord repair. Biomaterials.

[7] Nakajima H., Uchida K., Guerrero A.R., Watanabe S., Sugita D., Takeura N., Yoshida A., Long G., Wright K.T., Johnson W.E. (2012). Transplantation of mesenchymal stem cells promotes an alternative pathway of macrophage activation and functional recovery after spinal cord injury. J. Neurotrauma.

[8] Noort W.A., Feye D., Van Den Akker F., Stecher D., Chamuleau S.A., Sluijter J.P., Doevendans P.A. (2010). Mesenchymal stromal cells to treat cardiovascular disease: Strategies to improve survival and therapeutic results. Panminerva Med..

[9] Hare J.M., Traverse J.H., Henry T.D., Dib N., Strumpf R.K., Schulman S.P., Gerstenblith G., DeMaria A.N., Denktas A.E., Gammon R.S. (2009). A randomized, double-blind, placebo-controlled, dose-escalation study of intravenous adult human mesenchymal stem cells (prochymal) after acute myocardial infarction. J. Am. Coll. Cardiol..

[10] Zhu J., Liu Q., Jiang Y., Wu L., Xu G., Liu X. (2015). Enhanced angiogenesis promoted by human umbilical mesenchymal stem cell transplantation in stroked mouse is Notch1 signaling associated. Neuroscience.

[11] Xie N., Li Z., Adesanya T.M., Guo W., Liu Y., Fu M., Kilic A., Tan T., Zhu H., Xie X. (2016). Transplantation of placenta-derived mesenchymal stem cells enhances angiogenesis after ischemic limb injury in mice. J. Cell Mol. Med..

[12] Alexander J.K., Popovich P.G. (2009). Neuroinflammation in spinal cord injury: Therapeutic targets for neuroprotection and regeneration. Prog. Brain Res..

[13] Hausmann O.N. (2003). Post-traumatic inflammation following spinal cord injury. Spinal Cord.

[14] Popovich P.G., Guan Z., McGaughy V., Fisher L., Hickey W.F., Basso D.M. (2002). The neuropathological and behavioral consequences of intraspinal microglial/macrophage activation. J. Neuropathol. Exp. Neurol..

[15] Hagg T., Oudega M. (2006). Degenerative and spontaneous regenerative processes after spinal cord injury. J. Neurotrauma.

[16] Nandoe Tewarie R.D., Hurtado A., Ritfeld G.J., Rahiem S.T., Wendell D.F., Barroso M.M., Grotenhuis J.A., Oudega M. (2009). Bone marrow stromal cells elicit tissue sparing after acute but not delayed transplantation into the contused adult rat thoracic spinal cord. J. Neurotrauma.

[17] Chang J., Koh A.J., Roca H., McCauley L.K. (2015). Juxtacrine interaction of macrophages and bone marrow stromal cells induce interleukin-6 signals and promote cell migration. Bone Res..

[18] Fasslrinner F., Wobus M., Duryagina R., Müller K., Stopp S., Wehner R., Rauner M., Hofbauer L.C., Schmitz M., Bornhäuser M. (2012). Differential effects of mixed lymphocyte reaction supernatant on human mesenchymal stromal cells. Exp. Hematol..

[19] Ma S., Xie N., Li W., Yuan B., Shi Y., Wang Y. (2014). Immunobiology of mesenchymal stem cells. Cell Death Differ..

[20] Miceli V., Bulati M., Iannolo G., Zito G., Gallo A., Conaldi P.G. (2021). Therapeutic Properties of Mesenchymal Stromal/Stem Cells: The Need of Cell Priming for Cell-Free Therapies in Regenerative Medicine. Int. J. Mol. Sci..

[21] Lim J.-Y., Kim B.-S., Ryu D.-B., Kim T.W., Park G., Min C.-K. (2021). The therapeutic efficacy of mesenchymal stromal cells on experimental colitis was improved by the IFN-γ and poly (I: C) priming through promoting the expression of indoleamine 2, 3-dioxygenase. Stem Cell Res. Ther..

[22] Plotnikov E.Y., Pulkova N.V., Pevzner I.B., Zorova L.D., Silachev D.N., Morosanova M.A., Sukhikh G.T., Zorov D.B. (2013). Inflammatory pre-conditioning of mesenchymal multipotent stromal cells improves their immunomodulatory potency in acute pyelonephritis in rats. Cytotherapy.

[23] Zimmermann J.A., McDevitt T.C. (2014). Pre-conditioning mesenchymal stromal cell spheroids for immunomodulatory paracrine factor secretion. Cytotherapy.

[24] Li J.H., Zhang N., Wang J.A. (2008). Improved anti-apoptotic and anti-remodeling potency of bone marrow mesenchymal stem cells by anoxic pre-conditioning in diabetic cardiomyopathy. J. Endocrinol. Investig..

[25] Esmaeili R., Darbandi-Azar A., Sadeghpour A., Majidzadeh A.K., Eini L., Jafarbeik-Iravani N., Hoseinpour P., Vajhi A., Bakhshaiesh T.O., Masoudkabir F. (2021). Mesenchymal stem cells Pretreatment with stromal-derived factor-1 alpha augments cardiac function and angiogenesis in infarcted myocardium. Am. J. Med. Sci..

[26] Carvalho J.L., Braga V.B., Melo M.B., Campos A.C., Oliveira M.S., Gomes D.A., Ferreira A.J., Santos R.A., Goes A.M. (2013). Priming mesenchymal stem cells boosts stem cell therapy to treat myocardial infarction. J. Cell Mol. Med..

[27] Maldonado-Lasunción I., O’Neill N., Umland O., Verhaagen J., Oudega M. (2021). Macrophage-Derived Inflammation Induces a Transcriptome Makeover in Mesenchymal Stromal Cells Enhancing Their Potential for Tissue Repair. Int. J. Mol. Sci..

[28] Crisostomo P.R., Wang Y., Markel T.A., Wang M., Lahm T., Meldrum D.R. (2008). Human mesenchymal stem cells stimulated by TNF-alpha, LPS, or hypoxia produce growth factors by an NF kappa B- but not JNK-dependent mechanism. Am. J. Physiol. Cell Physiol..

[29] Ritfeld G.J., Nandoe Tewarie R.D., Vajn K., Rahiem S.T., Hurtado A., Wendell D.F., Roos R.A., Oudega M. (2012). Bone marrow stromal cell-mediated tissue sparing enhances functional repair after spinal cord contusion in adult rats. Cell Transplant..

[30] Mosser D.M., Edwards J.P. (2008). Exploring the full spectrum of macrophage activation. Nat. Rev. Immunol..

[31] Porcheray F., Viaud S., Rimaniol A.C., Leone C., Samah B., Dereuddre-Bosquet N., Dormont D., Gras G. (2005). Macrophage activation switching: An asset for the resolution of inflammation. Clin. Exp. Immunol..

[32] Basso D.M., Beattie M.S., Bresnahan J.C. (1996). Graded histological and locomotor outcomes after spinal cord contusion using the NYU weight-drop device versus transection. Exp. Neurol..

[33] Basso D.M., Beattie M.S., Bresnahan J.C. (1995). A sensitive and reliable locomotor rating scale for open field testing in rats. J. Neurotrauma.

[34] Kunkel-Bagden E., Dai H.-N., Bregman B.S. (1992). Recovery of function after spinal cord hemisection in newborn and adult rats: Differential effects on reflex and locomotor function. Exp. Neurol..

[35] Chaplan S.R., Bach F., Pogrel J., Chung J., Yaksh T. (1994). Quantitative assessment of tactile allodynia in the rat paw. J. Neurosci. Methods.

[36] Crowe M.J., Bresnahan J.C., Shuman S.L., Masters J.N., Beattie M.S. (1997). Apoptosis and delayed degeneration after spinal cord injury in rats and monkeys. Nat. Med..

[37] McTigue D.M., Tani M., Krivacic K., Chernosky A., Kelner G.S., Maciejewski D., Maki R., Ransohoff R.M., Stokes B.T. (1998). Selective chemokine mRNA accumulation in the rat spinal cord after contusion injury. J. Neurosci. Res..

[38] Fleming J.C., Norenberg M.D., Ramsay D.A., Dekaban G.A., Marcillo A.E., Saenz A.D., Pasquale-Styles M., Dietrich W.D., Weaver L.C. (2006). The cellular inflammatory response in human spinal cords after injury. Brain.

[39] Neri S., Borzi R.M. (2020). Molecular Mechanisms Contributing to Mesenchymal Stromal Cell Aging. Biomolecules.

[40] Jakovljevic J., Harrell C.R., Fellabaum C., Arsenijevic A., Jovicic N., Volarevic V. (2018). Modulation of autophagy as new approach in mesenchymal stem cell-based therapy. Biomed. Pharmacother..

[41] Gray A., Schloss R.S., Yarmush M. (2016). Donor variability among anti-inflammatory pre-activated mesenchymal stromal cells. Technology.

[42] Shelke G.V., Jagtap J.C., Kim D.-K., Shah R.D., Das G., Shivayogi M., Pujari R., Shastry P. (2018). TNF-α and IFN-γ together up-regulates par-4 expression and induce apoptosis in human neuroblastomas. Biomedicines.

[43] Cavaillon J. (1994). Cytokines and macrophages. Biomed. Pharmacother..

[44] Darwich L., Coma G., Peña R., Bellido R., Blanco E.J., Este J.A., Borras F.E., Clotet B., Ruiz L., Rosell A. (2009). Secretion of interferon-γ by human macrophages demonstrated at the single-cell level after costimulation with interleukin (IL)-12 plus IL-18. Immunology.

[45] Klusch A., Gorzelanny C., Reeh P.W., Schmelz M., Petersen M., Sauer S.K. (2018). Local NGF and GDNF levels modulate morphology and function of porcine DRG neurites, In Vitro. PLoS ONE.

[46] Fine E.G., Decosterd I., Papaloïzos M., Zurn A.D., Aebischer P. (2002). GDNF and NGF released by synthetic guidance channels support sciatic nerve regeneration across a long gap. Eur. J. Neurosci..

[47] Chen J., Chu Y., Chen J., Li B. (2010). Synergistic effects of NGF, CNTF and GDNF on functional recovery following sciatic nerve injury in rats. Adv. Med Sci..

[48] Widenfalk J., Lundströmer K., Jubran M., Brené S., Olson L. (2001). Neurotrophic factors and receptors in the immature and adult spinal cord after mechanical injury or kainic acid. J. Neurosci..

[49] Zhang L., Ma Z., Smith G.M., Wen X., Pressman Y., Wood P.M., Xu X.M. (2009). GDNF-enhanced axonal regeneration and myelination following spinal cord injury is mediated by primary effects on neurons. Glia.

[50] Le Blanc K., Mougiakakos D. (2012). Multipotent mesenchymal stromal cells and the innate immune system. Nat. Rev. Immunol..

[51] Tomchuck S.L., Zwezdaryk K.J., Coffelt S.B., Waterman R.S., Danka E.S., Scandurro A.B. (2008). Toll-like receptors on human mesenchymal stem cells drive their migration and immunomodulating responses. Stem Cells.

[52] Waterman R.S., Tomchuck S.L., Henkle S.L., Betancourt A.M. (2010). A new mesenchymal stem cell (MSC) paradigm: Polarization into a pro-inflammatory MSC1 or an Immunosuppressive MSC2 phenotype. PLoS ONE.

[53] Delarosa O., Dalemans W., Lombardo E. (2012). Toll-like receptors as modulators of mesenchymal stem cells. Front. Immunol..

[54] Romieu-Mourez R., François M., Boivin M.N., Bouchentouf M., Spaner D.E., Galipeau J. (2009). Cytokine modulation of TLR expression and activation in mesenchymal stromal cells leads to a proinflammatory phenotype. J. Immunol..

[55] Papatheodorou A., Stein A., Bank M., Sison C.P., Gibbs K., Davies P., Bloom O. (2017). High-Mobility Group Box 1 (HMGB1) Is Elevated Systemically in Persons with Acute or Chronic Traumatic Spinal Cord Injury. J. Neurotrauma.

[56] Blight A.R. (1992). Macrophages and inflammatory damage in spinal cord injury. J. Neurotrauma.

[57] Kopper T.J., McFarlane K.E., Bailey W.M., Orr M.B., Zhang B., Gensel J.C. (2019). Delayed Azithromycin Treatment Improves Recovery After Mouse Spinal Cord Injury. Front. Cell Neurosci..

[58] Bin S., Zhou N.F., Pan J., Pan F.M., Wu X.F., Zhou Z.H. (2017). Nano-carrier mediated co-delivery of methyl prednisolone and minocycline for improved post-traumatic spinal cord injury conditions in rats. Drug Dev. Ind. Pharm..

[59] Cheung T.S., Galleu A., von Bonin M., Bornhäuser M., Dazzi F. (2019). Apoptotic mesenchymal stromal cells induce prostaglandin E2 in monocytes: Implications for the monitoring of mesenchymal stromal cell activity. Haematologica.

[60] Galleu A., Riffo-Vasquez Y., Trento C., Lomas C., Dolcetti L., Cheung T.S., von Bonin M., Barbieri L., Halai K., Ward S. (2017). Apoptosis in mesenchymal stromal cells induces in vivo recipient-mediated immunomodulation. Sci. Transl. Med..

[61] Choudhery M.S., Khan M., Mahmood R., Mohsin S., Akhtar S., Ali F., Khan S.N., Riazuddin S. (2012). Mesenchymal stem cells conditioned with glucose depletion augments their ability to repair-infarcted myocardium. J. Cell. Mol. Med..

[62] Lutton C., Young Y.W., Williams R., Meedeniya A.C., Mackay-Sim A., Goss B. (2012). Combined VEGF and PDGF treatment reduces secondary degeneration after spinal cord injury. J. Neurotrauma.

[63] Oudega M. (2012). Molecular and cellular mechanisms underlying the role of blood vessels in spinal cord injury and repair. Cell Tissue Res..

[64] Ballabh P., Hu F., Kumarasiri M., Braun A., Nedergaard M. (2005). Development of tight junction molecules in blood vessels of germinal matrix, cerebral cortex, and white matter. Pediatr. Res..

[65] Tornavaca O., Chia M., Dufton N., Almagro L.O., Conway D.E., Randi A.M., Schwartz M.A., Matter K., Balda M.S. (2015). ZO-1 controls endothelial adherens junctions, cell–cell tension, angiogenesis, and barrier formation. J. Cell Biol..

[66] De Becker A., Riet I.V. (2016). Homing and migration of mesenchymal stromal cells: How to improve the efficacy of cell therapy?. World J. Stem Cells.

[67] Zachar L., Bačenková D., Rosocha J. (2016). Activation, homing, and role of the mesenchymal stem cells in the inflammatory environment. J. Inflamm. Res..

[68] Caffi V., Espinosa G., Gajardo G., Morales N., Durán M.C., Uberti B., Morán G., Plaza A., Henríquez C. (2020). Pre-conditioning of Equine Bone Marrow-Derived Mesenchymal Stromal Cells Increases Their Immunomodulatory Capacity. Front. Vet. Sci..

[69] Rafat A., Mohammadi Roushandeh A., Alizadeh A., Hashemi-Firouzi N., Golipoor Z. (2019). Comparison of The Melatonin Preconditioning Efficacy between Bone Marrow and Adipose-Derived Mesenchymal Stem Cells. Cell J..

[70] Pourjafar M., Saidijam M., Mansouri K., Ghasemibasir H., Karimi Dermani F., Najafi R. (2017). All-trans retinoic acid preconditioning enhances proliferation, angiogenesis and migration of mesenchymal stem cell in vitro and enhances wound repair in vivo. Cell Prolif..

[71] Tsai L.K., Wang Z., Munasinghe J., Leng Y., Leeds P., Chuang D.M. (2011). Mesenchymal stem cells primed with valproate and lithium robustly migrate to infarcted regions and facilitate recovery in a stroke model. Stroke.

[72] Sheng H., Wang Y., Jin Y., Zhang Q., Zhang Y., Wang L., Shen B., Yin S., Liu W., Cui L. (2008). A critical role of IFNgamma in priming MSC-mediated suppression of T cell proliferation through up-regulation of B7-H1. Cell Res..

[73] Freytes D.O., Kang J.W., Marcos-Campos I., Vunjak-Novakovic G. (2013). Macrophages modulate the viability and growth of human mesenchymal stem cells. J. Cell. Biochem..

[74] Maldonado-Lasuncion I., Verhaagen J., Oudega M. (2018). Mesenchymal Stem Cell-Macrophage Choreography Supporting Spinal Cord Repair. Neurotherapeutics.

[75] Siegenthaler M.M., Tu M.K., Keirstead H.S. (2007). The extent of myelin pathology differs following contusion and transection spinal cord injury. J. Neurotrauma.

