# Supplementary material for: The Effect of Inflammatory Priming on the Therapeutic Potential of Mesenchymal Stromal Cells for Spinal Cord Repair
Source: Cells. 2021 May 25;10(6):1316. doi: 10.3390/cells10061316 (PMC8227154; doi:10.3390/cells10061316)
Supplement: Supplementary file 1 [file cells-10-01316-s001.zip › cells-1187900-supplementary.pdf]

Supplementary Information

The effect of inflammatory priming on the therapeutic potential of mesenchymal stromal cells for spinal cord repair

Ines Maldonado-Lasunción [1–4,*], Agnes E. Haggerty [2], Akinori Okuda [2,5], Tokumitsu Mihara [2,6], Natalia de la Oliva [2], Joost Verhaagen [1] and Martin Oudega [3,4,7,8*]

List of contents:

I. Table S1: Primary antibodies used in this study
II. Table S2. Secondary antibodies used in this study
III. Figure S1: Macrophage inflammatory phenotype over time
IV. Figure S2: Blood vessel presence in the rostral, central and caudal regions of the injury
V. Figure S3: Hind limb sensorimotor performance

I. Table S1. Primary antibodies used in this study

Antibody	Reactivity	Manufacturer	Catalog number	Concentration	Triton*
Rabbit anti-GFP	Rat	Millipore	AB3080P	1:1000	Yes
Mouse anti-GFAP	Rat	Sigma	G3893	1:500	Yes
Rabbit anti-NeuN	Rat	Millipore	ABN78	1:500	Yes
Mouse anti-NF	Rat	Hybridoma	Made in-house	1:3 (Conditioned medium)	Yes
Mouse anti-ED1	Rat	Millipore	MAB1435	1:200	No
Rabbit anti-CD86	Rat	Abcam	Ab53004	1:500	No
Rabbit anti-CD206	Rat	Abcam	Ab64693	1:500	No
Rabbit anti-CD163	Rat	Abcam	Ab182422	1:500	No
Mouse anti-RECA-1	Rat	BioRad	MCA970GA	1:200	No
Mouse anti-Occludin	Rat	Invitrogen	331500	1:250	Yes
Rabbit anti-ZO-1	Rat	Invitrogen	402200	1:100	Yes

* This column indicates whether the blocking solution contained Triton X100 (*i.e.* for intracellular stainings) or not (*i.e.* for extracellular stainings).

II. Table S2. Secondary antibodies used in this study

Primary target	Antibody	Conjugate	Manufacturer	Catalog number	Concentration
GFP	Goat anti-rabbit	AF-488	ThermoFisher	A11008	1:500
GFAP	Goat anti-mouse	AF-555	Abcam	Ab150114	1:500
NeuN	Goat anti-rabbit	AF-647	ThermoFisher	A21245	1:500
NF	Goat anti-mouse	AF-555	Abcam	Ab150114	1:500
ED1	Goat anti-mouse	AF-647	ThermoFisher	A21236	1:500
CD86	Goat anti-rabbit	AF-555	ThermoFisher	A21428	1:500
CD206	Goat anti-rabbit	AF-555	ThermoFisher	A21428	1:500
CD163	Goat anti-rabbit	AF-555	ThermoFisher	A21428	1:500
RECA-1	Goat anti-mouse	AF-555	Abcam	Ab150114	1:500
Occludin	Goat anti-mouse	AF-555	Abcam	Ab150114	1:500
ZO-1	Goat anti-rabbit	AF-647	ThermoFisher	A21245	1:500

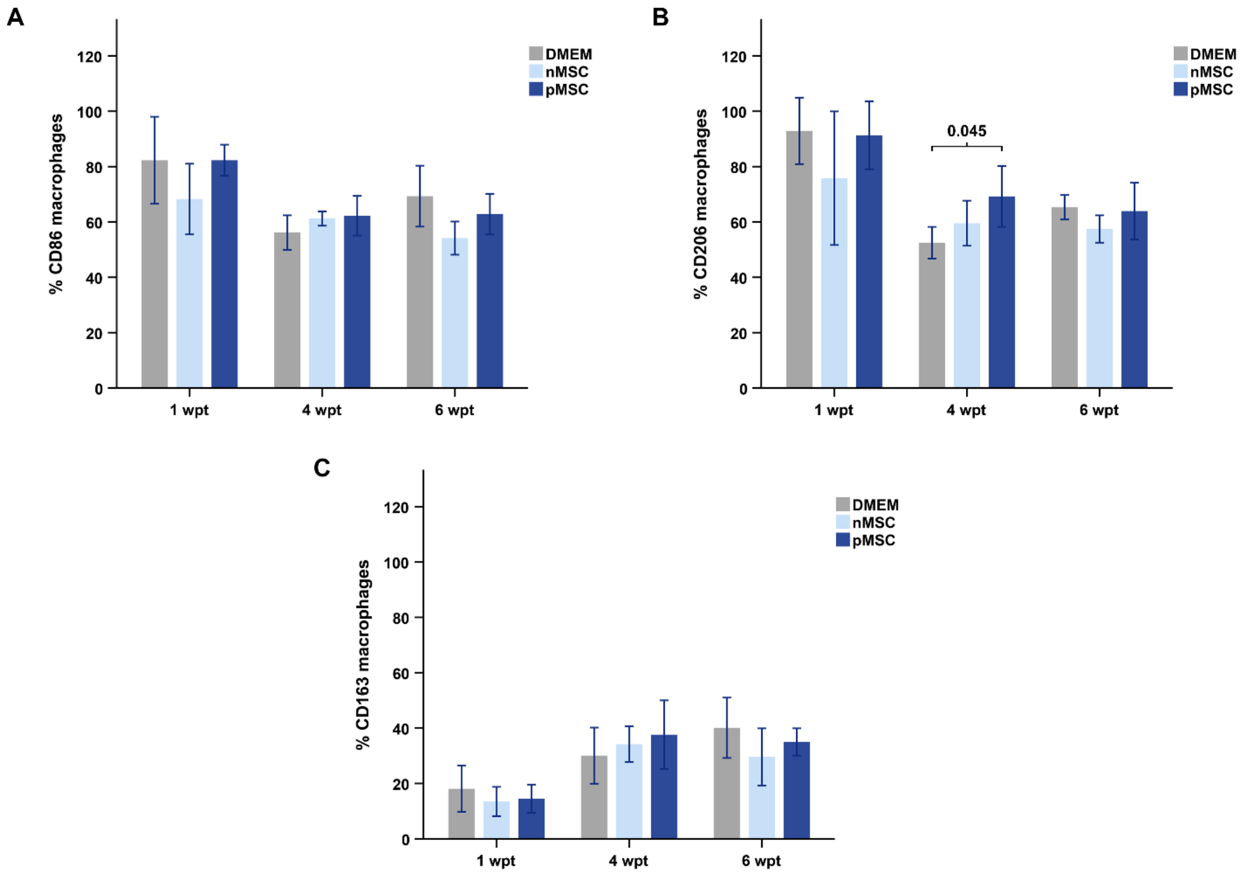

III. **Figure S1. Evolution of macrophage phenotype over time.** The percentage of each surface marker was calculated from the total of macrophages (ED1 positive) found in the injury site for each experimental group at 1, 4 and 6 wpt. **A.** Percentage of pro-inflammatory macrophages, positive for CD86. **B.** Percentage of early acting anti-inflammatory macrophages, positive for CD206. **C.** Percentage of late acting anti-inflammatory macrophages, positive for CD163. Bars and error bars represent the mean and SEM, respectively (*N* = 6 per group and time point.). Abbreviations: wpt: weeks post transplantation; DMEM: Dulbecco's Modified Eagle's Medium (vehicle control); nMSC: naïve MSC; pMSC: primed MSC; MSC: mesenchymal stromal cell.

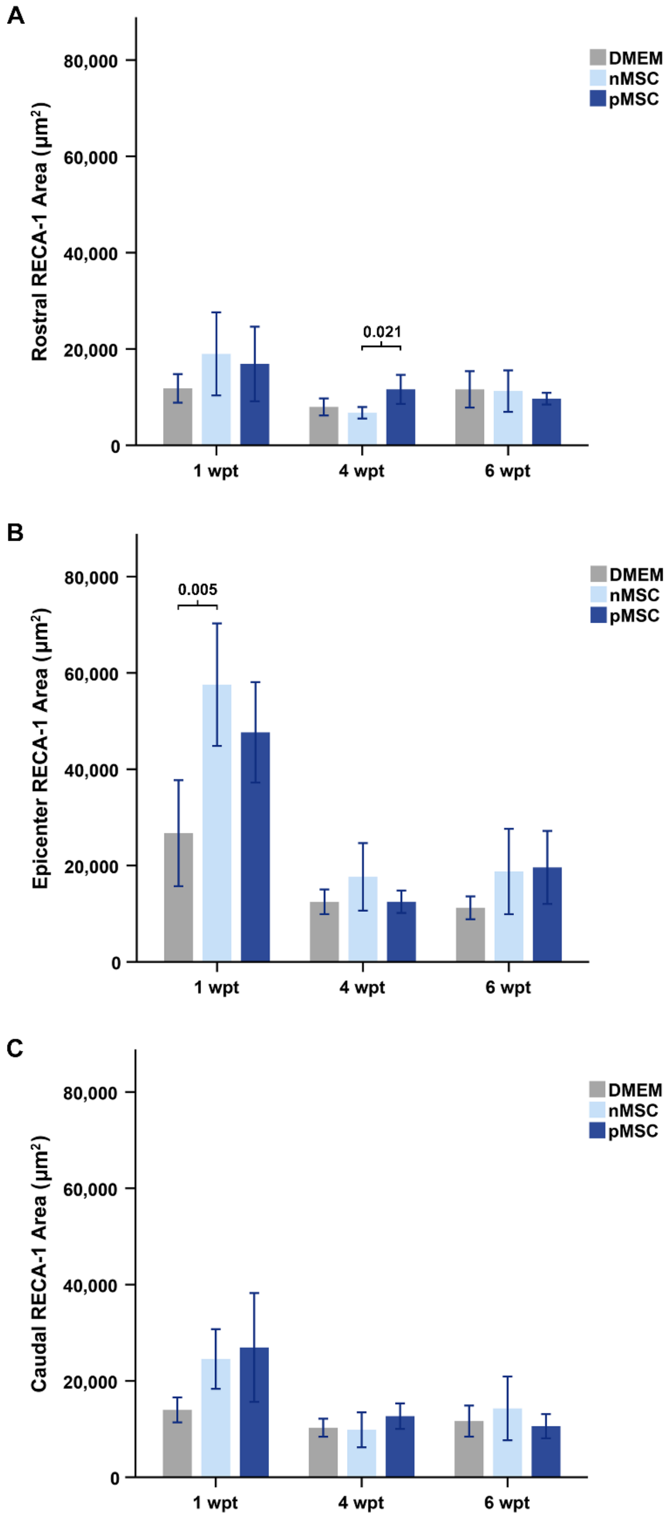

IV. **Figure S2. Blood vessel presence in and surrounding the injury site over time.** The area of RECA-1 staining was calculated in the areas just rostral (**A**), central (**B**) and caudal (**C**) to the injury for each experimental group at 1, 4 and 6 wpt. Bars and error bars represent

the mean and SEM, respectively (N = 6 per group and time point.). Abbreviations: RECA-1: rat endothelial cell antigen 1; wpt: weeks post transplantation; DMEM: Dulbecco's Modified Eagle's Medium (vehicle control); nMSC: naïve MSC; pMSC: primed MSC; MSC: mesenchymal stromal cell.

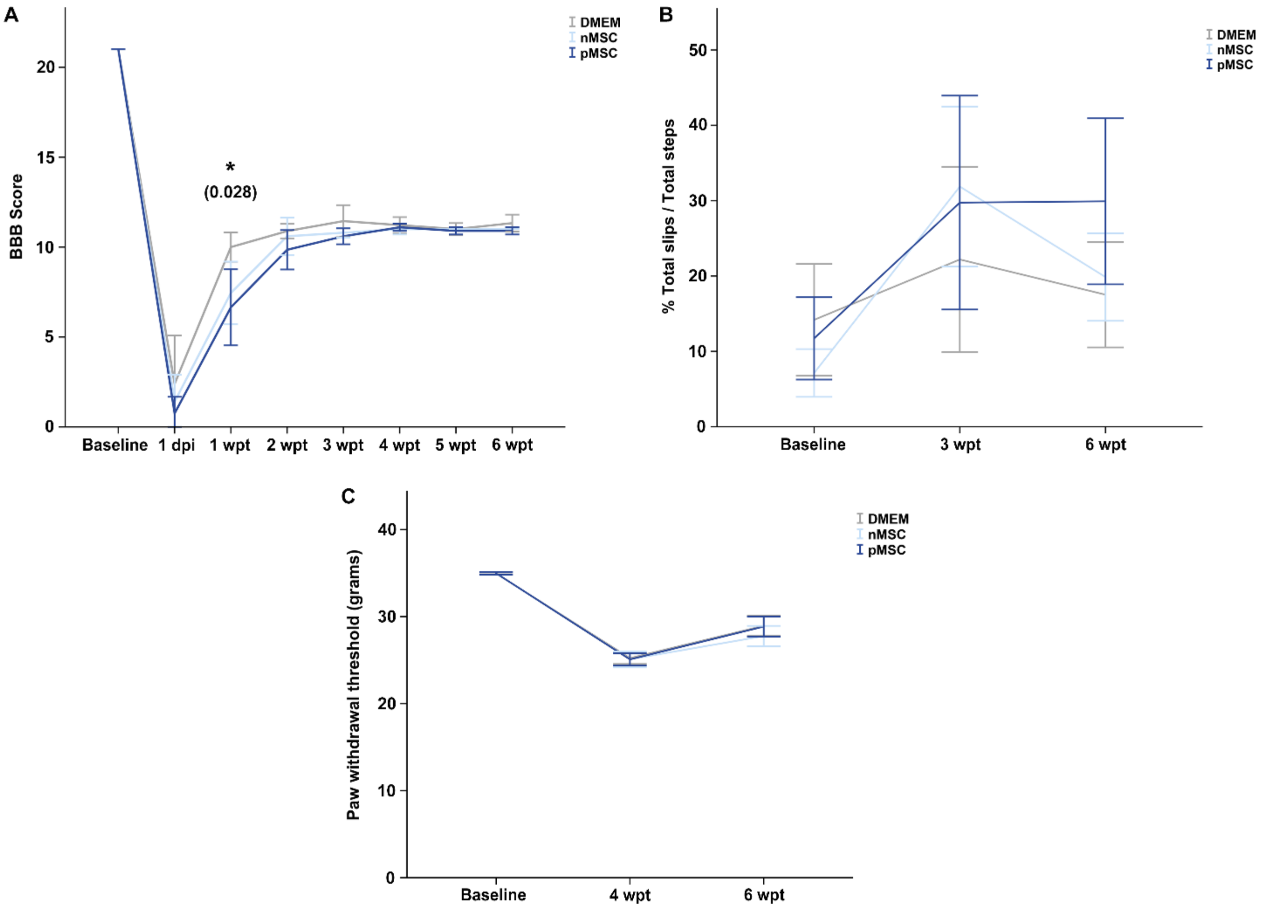

V. **Figure S3. Locomotor and sensory performance was similar between all experimental groups over time.** (**A**) Overground walking ability improved similarly for all groups after SCI and transplantation. The graph represents the BBB locomotor scores before SCI (baseline), one day after SCI (1 dpi) and every week after transplantation at 3 days post-injury, up to 6 weeks. (**B**) Sensorimotor performance was similar among experimental groups across time, as shown by the percentage of slips on the horizontal ladder test. The graph represents the percentage of total steps that resulted in a slip at baseline and 3 and 6 weeks after transplantation. (**C**) Sensitivity to mechanical stimulation was similar between groups at baseline and 4 and 6 weeks after transplantation, as seen by the Von Frey hypersensitivity test. Points and error bars represent the mean and SEM, respectively (N = 10 per group and time point.). Abbreviations: DMEM: Dulbecco's Modified Eagle's Medium (vehicle control); nMSC: naïve MSC; pMSC: primed MSC; MSC: mesenchymal stromal cell; dpi: days post injury; wpt: weeks post transplant.
